# Supplementary figures and images for: Dynamics of IgM and IgG responses to the next generation of engineered Duffy binding protein II immunogen: Strain-specific and strain-transcending immune responses over a nine-year period
Source: PLoS One. 2020 May 7;15(5):e0232786. doi: 10.1371/journal.pone.0232786 (PMC7205269; doi:10.1371/journal.pone.0232786)

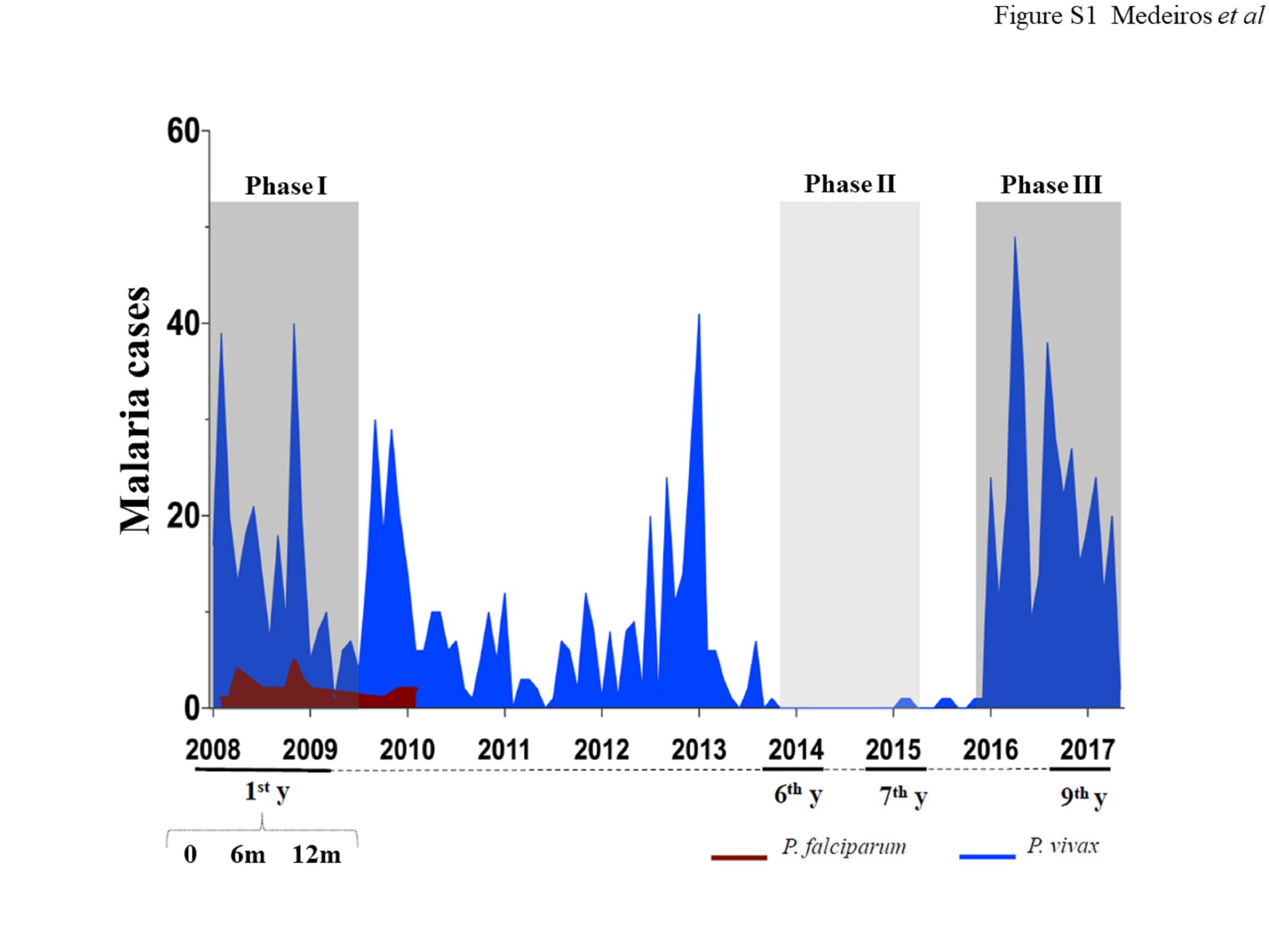

Supplement: S1 Fig — P. vivax (blue) and P. falciparum (red) microscopy diagnosed case report data in Rio Pardo were provided by the National Malaria Surveillance Information System (SIVEP-Malaria) and plotted per month. The longitudinal study comprises six cross-sectional surveys during 2008–2017, which includes periods of high (dark-grey, phase I and III) and low (light-grey, phase II) malaria transmission; the first three cross-sectional surveys were carried-out during the first year (baseline, 6 and 12 months); three carried-out 6th, 7th and 9th years later. Modified from Pires et al., 2018 [33]. (TIF) [file pone.0232786.s001.tif]

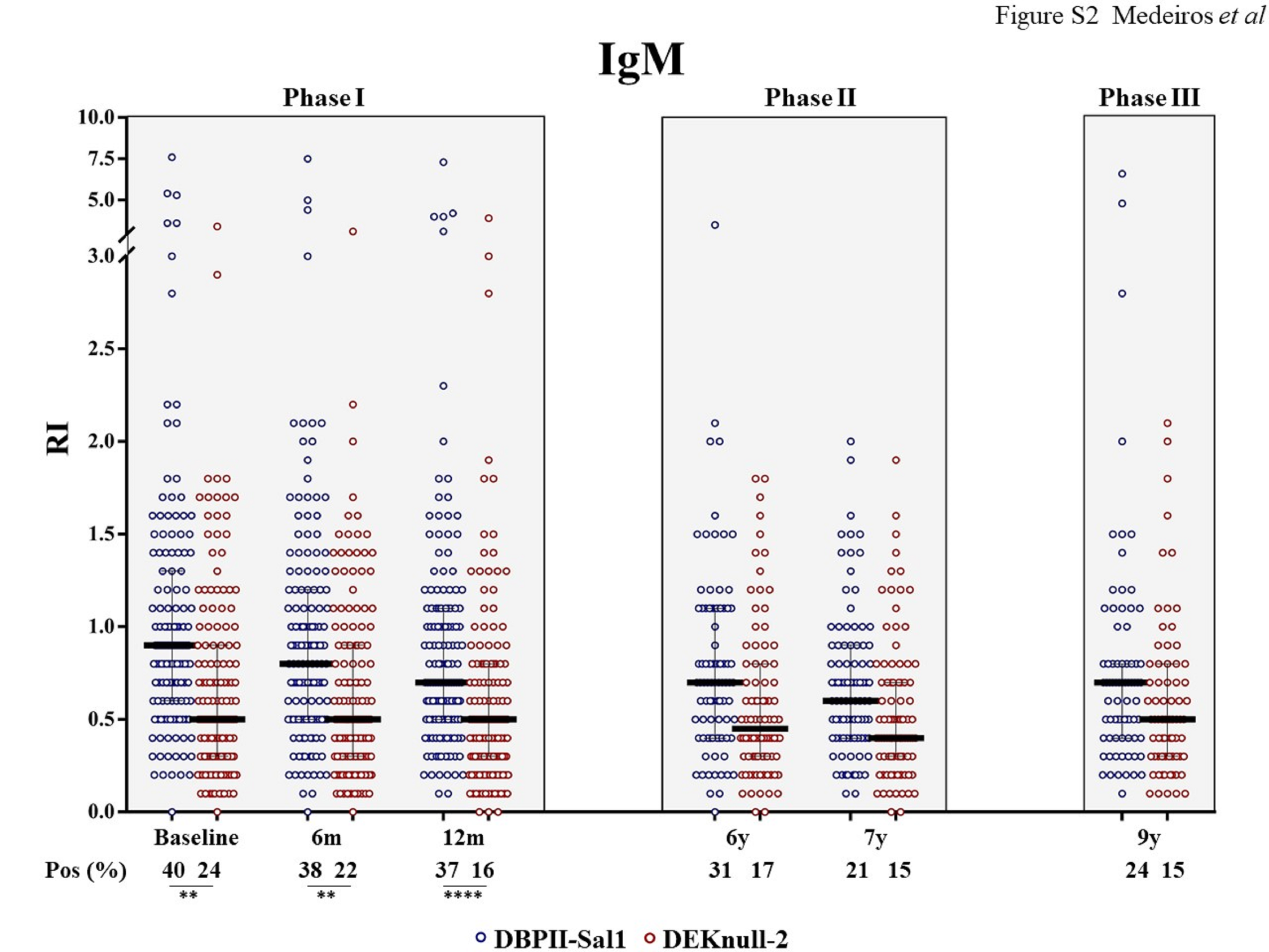

Supplement: S2 Fig — The IgM responses were expressed as Reactivity Index (RI), with Reactivity Index (RI)>1.0 considered positive. The individual values are represented by blue (DBPII-Sal1) and red (DEKnull-2) open circles. Transversal lines indicate medians and interquartile ranges. The cross-sectional surveys were carried-out as described in legend to S1 Fig. The frequency of seropositive subjects (Pos (%)) on each cross-sectional survey is represented below each graphic. Different number of asterisks indicate the variation on p value (*p< 0.05 to **p<0.0001; Fisher’s exact test), for significance differences between the frequency of DBPII-Sal1 and DEKnull-2 response. (TIF) [file pone.0232786.s002.tif]

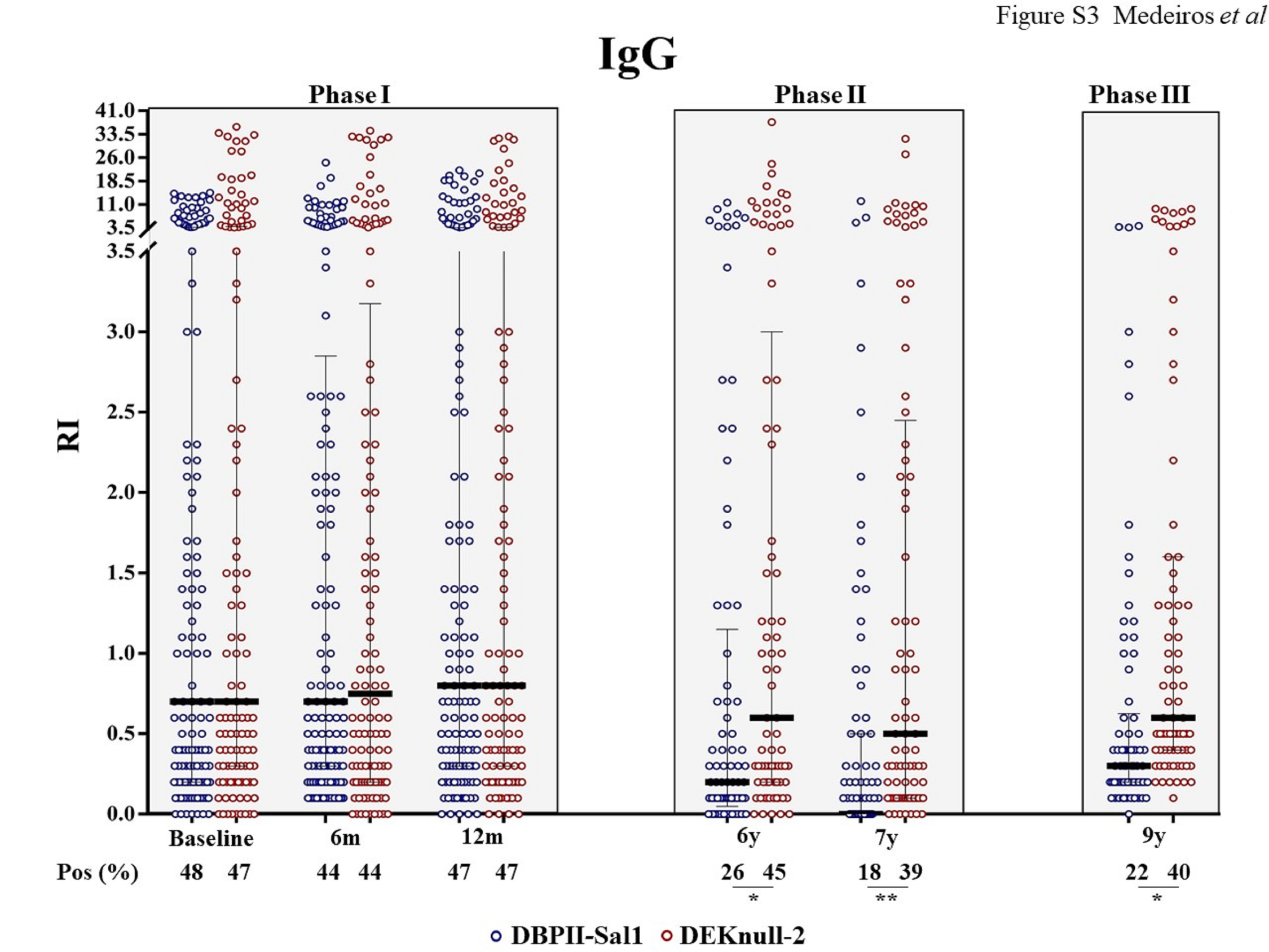

Supplement: S3 Fig — The IgG responses were expressed as Reactivity Index (RI), with Reactivity Index (RI)>1.0 considered positive. The individual values are represented by blue (DBPII-Sal1) and red (DEKnull-2) open circles. Transversal lines indicate medians and interquartile ranges. The cross-sectional surveys were carried-out as described in legend to S1 Fig, with IgG original data obtained from Pires et al., 2018 [33]. The frequency of seropositive subjects (Pos (%)) on each cross-sectional survey is represented below each graphic. Different number of asterisks indicates the variation on p value (*p< 0.05 to **p<0.0001; Fisher’s exact test), for significance differences between the frequency of DBPII-Sal1 and DEKnull-2 response. (TIF) [file pone.0232786.s003.tif]

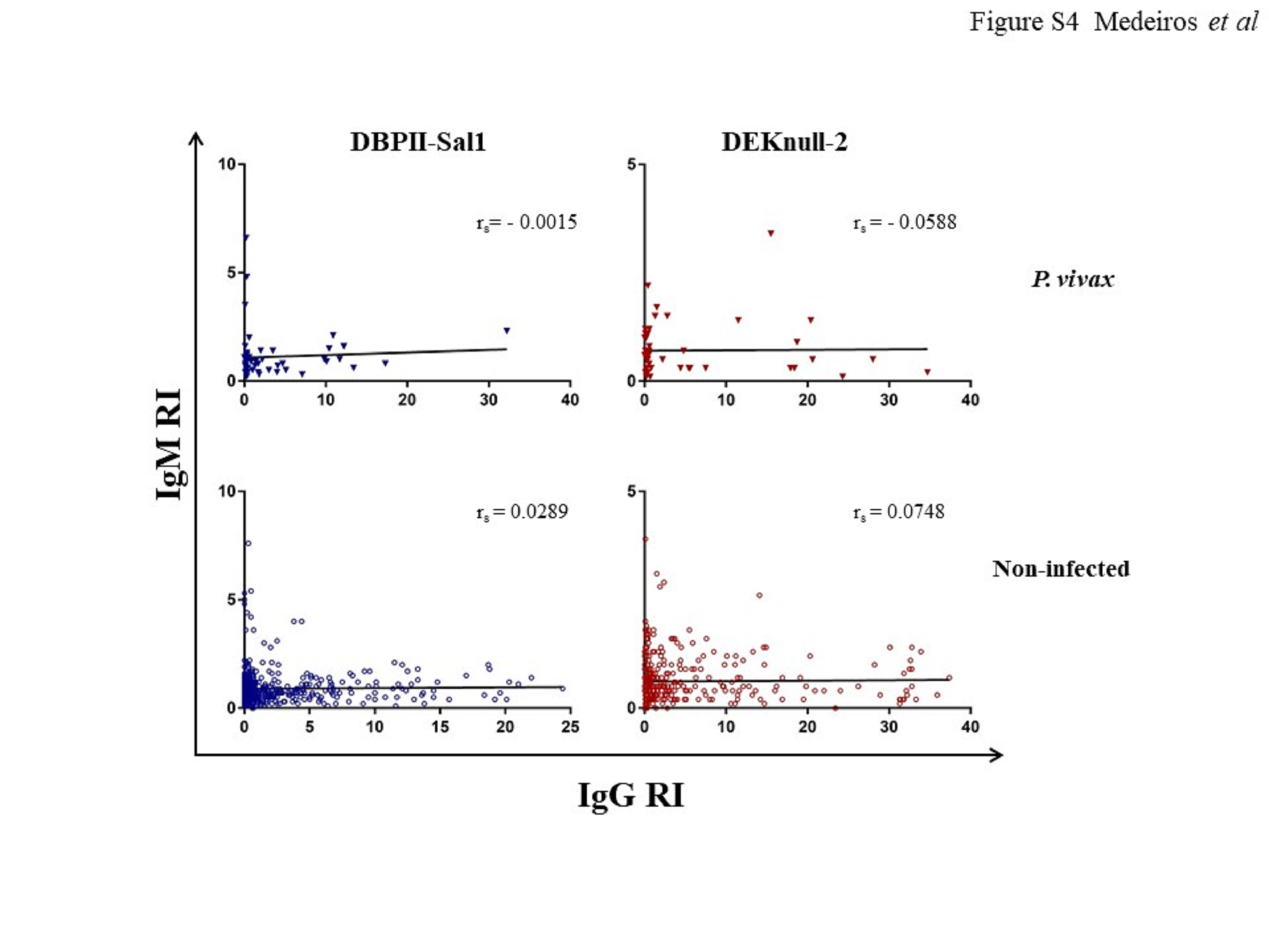

Supplement: S4 Fig — The correlation between IgM and IgG antibodies response were performed separately to subjects with acute P. vivax infections (closed triangle) and non-infected individuals (open circles) to each protein. (TIF) [file pone.0232786.s004.tif]
