# Supplementary material for: Dynamics of IgM and IgG responses to the next generation of engineered Duffy binding protein II immunogen: Strain-specific and strain-transcending immune responses over a nine-year period
Source: PLoS One. 2020 May 7;15(5):e0232786. doi: 10.1371/journal.pone.0232786 (PMC7205269; doi:10.1371/journal.pone.0232786)
Supplement: S1 Table — (DOCX) [file pone.0232786.s005.docx]

| \| **S1 Table 1. Levels of IgM and IgG antibodies response against *P. vivax* DBPII*-*proteins during the 9 years follow-up study.** \| \| \| \| \| \| \| \| \| \| \| --- \| --- \| --- \| --- \| --- \| --- \| --- \| --- \| --- \| --- \| \|  \|  \| **Phase I** \| \| \|  \| **Phase II** \| \|  \| **Phase III** \| \|  \|  \| **Baseline**  Median (IQR)¹ \| **6 months**  Median (IQR)¹ \| **12 months**  Median (IQR)¹ \|  \| **6 years**  Median (IQR)¹ \| **7 years**  Median (IQR)¹ \|  \| **9 years**  Median (IQR)¹ \| \| **IgM** \| DBPII-Sal1  DEKnull-2 \| 0.9 (0.6-1.3)  *vs*  0.5 (0.3-0.9) \| 0.8 (0.5-1.2)  *vs*  0.5 (0.3-0.9) \| 0.7 (0.5-1.1)  *vs*  0.5 (0.3-0.8) \|  \| 0.7 (0.4-1.1)  *vs*  0.5 (0.3-0.8) \| 0.6 (0.4-0.9)  *vs*  0.4 (0.3-0.7) \|  \| 0.7 (0.4-0.8)  *vs*  0.5 (0.3-0.8) \| \|  \|  \| \|  \|  \| \| **p value^2^** \| **<0.0001** \| **<0.0001** \| **<0.0001** \|  \| **0.0008** \| **0.0013** \|  \| **0.0154** \| \| **IgG** \| DBPII-Sal1  DEKnull-2 \| 0.7 (0.2-3.7)  *vs*  0.7 (0.3-4.0) \| 0.7 (0.3-2.9)  *vs*  0.8 (0.2-3.2) \| 0.8 (0.3-3.7)  *vs*  0.8 (0.3-3.7) \|  \| 0.2 (0.1-1.2)  *vs*  0.6 (0.2-3.0) \| 0.0 (0.0-0.5)  *vs*  0.5 (0.1-2.5) \|  \| 0.3 (0.2-0.7)  *vs*  0.6 (0.4-1.6) \| \|  \|  \| \|  \|  \| \| **p value^2^** \| **0.4758** \| **0.9343** \| **0.8528** \|  \| **0.0003** \| **<0.0001** \|  \| **<0.0001** \| \| ^1^Median and InterQuartile Range (IQR) of Reactivity Index (RI) determined by conventional ELISA from serum of *P. vivax*-exposed individuals. ELISA was performed with recombinants proteins and RI was defined as the ratio between sample mean OD and cut-off mean OD. Cut-off was calculated as the mean OD from 20 non-exposed volunteers plus two standard deviations.  ^2^p value was obtained by Mann-Whitney statistical analysis. \| \| \| \| \| \| \| \| \| \| |
| --- | --- | --- | --- | --- | --- | --- | --- | --- | --- | --- | --- | --- | --- | --- | --- | --- | --- | --- | --- | --- | --- | --- | --- | --- | --- | --- | --- | --- | --- | --- | --- | --- | --- | --- | --- | --- | --- | --- | --- | --- | --- | --- | --- | --- | --- | --- | --- | --- | --- | --- | --- | --- | --- | --- | --- | --- | --- | --- | --- | --- | --- | --- | --- | --- | --- | --- | --- | --- | --- | --- | --- | --- | --- | --- | --- | --- | --- | --- | --- | --- | --- | --- | --- | --- | --- | --- |
