# Supplementary material for: Dynamics of IgM and IgG responses to the next generation of engineered Duffy binding protein II immunogen: Strain-specific and strain-transcending immune responses over a nine-year period
Source: PLoS One. 2020 May 7;15(5):e0232786. doi: 10.1371/journal.pone.0232786 (PMC7205269; doi:10.1371/journal.pone.0232786)
Supplement: Attachment — Submitted filename: Medeiros_rebuttal_letter_PlosNeglect_to_PlosOne .pdf [file pone.0232786.s006.pdf]

Ministério da Saúde
FIOCRUZ
Fundação Oswaldo Cruz
Instituto René Rachou
Laboratório de Malária
Belo Horizonte, MG, Brasil

PONE-D-20-02118
"Dynamics of IgM and IgG responses to the next generation of engineered Duffy binding protein II immunogen: strain-specific and strain-transcending immune responses over a nine-year period"

January 2020

To Editorial board
PLoS ONE

Rebuttal letter to points made by the Editor and/or reviewers of *PLoS Negl Trop Dis*

General comments

Based on the reviewers' comments, the editorial group of *PLoS Negl Trop Dis* recommended that the current manuscript should be more suitable for a more specialized journal such as PLoS ONE. Although we decided to follow the recommendation and submit it to PLoS One, we would like to clarify that *major concerns raised by PLoS Negl's reviewers were in the sense of identify/characterize the mechanism of long-term strain-specific IgM response, which was outside of the scope of the current MS*. To elucidate this type of immune response it will be required *(i)* to sequence different DBPII variants circulating in the study area for a period of at least 9-years (as the study involved long-term multiple serological cross-sectional surveys); *(ii)* to produce recombinant DBPII proteins corresponding to each of predominant variants circulating in the study area during the last 9-years; *(iii)* to identify/characterize subpopulation of memory B cells DBPII-specific (as this was a retrospective serological study PBMCs are not available). *Although relevant, all these suggestions made by the previous reviewers will demand an extraordinary amount of work that should be pursued in futures studies in this field of investigation.* Here, *we present serological data of a 9-years follow-up study* carried-out in a community of the Brazilian Amazon region, in which we sought to investigate the relationship between IgG and *the less studied IgM antibody response against conserved (DEKnull-2) and variable* DBPII epitopes *(Sal-1, a DBPII variant that is naturally circulating in the study area; please see specific comments below)*. We are confident *that our findings of long-term IgM response emphasize the need to understand the role of the IgM specific antibodies in both natural infection and vaccine antigens.*

Specific comments

Reviewer #1 ***"****This is an interesting study that involves multiple cross-sectional surveys. Overall, statistical analysis has been properly done. A few concerns remain, however, regarding the choice of antigens used to measure antibody levels. (...) it remains unclear whether the Sal-I variant is commonly found in locally circulating parasites. Sal-I was defined as the "natural DBPII variant circulating in the study area" but no data or reference is provided in support to this statement."*

Authors: Although we do not have DBPII sequence from all cross-sectional survey, our previous data confirm Sal-1 DBPII variant as highly prevalent in the study area (Kano et al., *Trop Med Int Health. 17(8):*989-1000, 2012), and frequent in the Brazilian Amazon area (Sousa et al., *Malar J. 9:*334, 2010; Nobrega de Sousa et al. *PLoS One. 6(8):*e22944, 2011). These findings are supported by the frequencies and levels of anti-Sa1 antibodies previously detected in the study area (Kano et al., *PLoS Negl Trop Dis.* 10(12):e0005177, 2016; Kano et al., *Sci Rep. 8(1):*13851, 2018). *Consequently, we are confident that Sal-1 did not represent a minor DBPII variant to which study subjects were exposed. Instead of that, Sal1 is a key DBPII variant to be used in the study area as a representative sequence of specie-specific immune response*. In the revised version of the MS, we clarified this topic (please see discussion section, highlighted text in the track changes version). Also, limitations of the study were addressed in the revised MS.

Reviewer #1 (…) Second, it remains unclear whether the recombinant antigens are correctly refolded (…)

DBPII production (according Ntumngia et al., 2012)
Authors: Our research group has a long-term collaboration with Dr. John H. Adams'group (USF, USA) who has expertise in the production/ refolded of DBPII recombinant proteins. To confirm the functional refolded, the refolded antigens were tested for their ability to bind Duffy-positive human erythrocytes in an erythrocyte-binding assay as previously described (Ntumngia et al., *Infect Immun., 80(3):*1203-8.2012). This information was included in the revised MS.

Reviewer #1 (…) no attempt was made to associate levels of anti-PvDBP antibodies (and, more specifically, levels of binding-inhibitory antibodies) with protection from clinical vivax malaria in the study population (…).

Authors: The current study was not design to evaluate clinical protection induced by DBPII inhibitory antibodies (BIAbs as detected by functional assays). First, because the main goal of the MS was to evaluate the relationship between IgM and IgG antibody responses as detected by ELISA, which is not an assay to evaluate protection. Here, a small group of individuals with BIAbs response was analyzed to investigate whether a stable DBPII-specific IgM response could interfere with the profile of BIAbs responder. Finally, our sample size precludes any conclusion about clinical protection, a topic that was outside of the scope of the MS.

Reviewer #2: This is primarily descriptive study (…). The paper is clearly written, the figures reasonable and informative and statistical analysis straightforward and appropriate.

Authors: Yes, we agree with the reviewer that this is a descriptive well-conducted cohort

study that was not design to investigate the mechanisms associates with the long-term IgM response (see general comments above). While scant longitudinal data are available about secondary IgM responses in P. vivax-exposed populations, the present study provides the first evaluation of the dynamic of IgM vs. IgG responses to a novel surface-engineered *P.vivax* DBPII-based vaccine. The reason for the persistence of DBPII-specific IgM response in our cohort merits further investigations.

Reviewer #2: (…) What is new in the current study is the examination of IgM response which seems to not vary as much over time or with overall Pv infection rates, and responses are consistently higher in the rPvDBPII compared to DEKnull variant. This is of interest, but it not explored further, e.g. are there persistent antigen-specific B cells (…).

Authors: As quoted before (see general comments), this is a retrospective serological follow-up study and no PBMCs were available from previous surveys. Further studies should identify /characterize subpopulation of memory B cells DBPII-specific (on going experiments). As inhibitory antibodies (BIAbs) evaluated here was performed against DBPII, future experiments should target BIAbs anti-DEKnull2. Limitations of the study were addressed in the revised version of MS.

Sincerely yours,

Luzia H Carvalho, Ph. D.

Flora S Kano, Ph.D.

Corresponding Authors
Malaria Molecular Biology and Immunology Unit (BMIM)
Instituto Rene Rachou, Fundação Oswaldo Cruz, FIOCRUZ, Brazilian Ministry of Health
Av. Augusto de Lima 1715, 30190-002 Belo Horizonte, MG, Brazil
Fax: 55-31-32953115,
Tel: 55-31-33497793
luzia.carvalho@fiocruz.br
flora.kano@fiocruz.br
