# Supplementary material for: Dynamics of IgM and IgG responses to the next generation of engineered Duffy binding protein II immunogen: Strain-specific and strain-transcending immune responses over a nine-year period
Source: PLoS One. 2020 May 7;15(5):e0232786. doi: 10.1371/journal.pone.0232786 (PMC7205269; doi:10.1371/journal.pone.0232786)
Supplement: Attachment — Submitted filename: Medeiros_etal_Response_to_Reviewers.docx [file pone.0232786.s007.docx]

***PONE-D-20-02118***
*Dynamics of IgM and IgG responses to the next generation of engineered Duffy binding protein II immunogen: strain-specific and strain-transcending immune responses over a nine-year period.*

**RESPONSES TO REVIEWERS**

**REVIEWER # 1**

**Major comment. *(…)*** *The authors demonstrate for the first time the presence of long-lasting IgM antibodies directed against PvDBPII (…) This is the first study to systematically analyze IgM and IgG against PvDBPII (…)the descriptive analysis presented here is interesting and important. (… )the analysis provides interesting insights into development of antibody responses against PvDBPII following natural exposure to P. vivax. The authors have responded to the queries raised earlier adequately.*

AUTHORS: We thank the reviewer for the careful examination of the manuscript and rebuttal letter, and to consider our work well carried-out and relevant for this field of investigation.

**REVIEWER # 2**

**Major comment**

**Rev#2 (…)** Overall, this manuscript is of interest, clearly written some points, informative and statistical analysis has been properly done (…). However, some of these findings had been reported using same field study from same group. And then it is required the authors to present these data with *clearly and carful editing to avoid misleading as a redundant publication* (…). *The major IgG response against DBPII-Sal1 & DEKnull-2 was reported on Ref 35,* fig 3.

AUTHORS: First of all, we thank the reviewer for the careful examination of our manuscript and consider the results relevant for this field of investigation. Regarding the concern of redundant publication, we must clarify that the MS from Pires et al (ref 35) was design to evaluate *the seroconversion rates as a tool for a rapid assessment of P.vivax malaria transmission intensity.* For that, we include blood-stage antigens covering a wide range of immunogenicity such as MSP1-19, AMA-1 and DBPII-related proteins. An unsupervised learning algorithm such as k-means clustering allowed categorizing individuals based on the magnitude and breadth (specificities for multiple antigens including AMA-1, DBPs and MSP-1) of their conventional antibody response. In that MS (ref 35), *we demonstrate that IgG antibodies against AMA-1/MSP1-19 were much more appropriate to detect temporal fluctuation of P.vivax transmission that the less immunogenic DBPII-related proteins*. Consequently, in the previous MS we investigated which antigen should be most appropriate for detecting fluctuations in *P.vivax* malaria transmission.

Aiming towards universal strain-transcending DBPII immunity, in the current MS, we evaluate for the first time the long-term DBPII-specific IgM response and the relationship of IgM antibodies with (i) conventional IgG response and their (ii) ability to blocking the interaction ligand-receptor (binding inhibitory activity/BIAbs, as detected by COS-7 assays). *Consequently, there is no overlap between the current MS and ref 35*. As scant longitudinal data are available about secondary IgM responses in P. vivax-exposed populations, we are confident that our findings fill a gap in the literature for providing the first description of the of the dynamic of IgM versus IgG responses to a novel surface-engineered *P.vivax* DBPII-based vaccine (*the relevance of studying IgM antibodies was included below; Major comment/Specific comment 1*).

Finally, we would like to emphasize that long-term follow-up studies are expensive, time consumer, and offer an exceptional opportunity to investigate unappreciated topics. Consequently, to focus on DBPII-specific IgM responses, we took advantage of the long-term follow-up study previously carried out in the Amazon rainforest, where different profiles of DBPII-specific IgG responders were identified (as described in ref 35). We are confident that the results presented here will contribute to further optimize the design of new DBPII immunogens such as DEKnull-2.

In the revised version of the MS, we clarified this concern raised by the reviewer (please see track changes copy, for example, in the last paragraph of introduction). Also we explained in the legend of Fig 3 (now S3 Fig, as requested by the reviewer) that IgG original data were obtained from Pires et al., 2018 (previous ref 35, and in the revised version ref 33).

***Rev#2:*** ***Major comment -Rev#2:*** (…)*it is still unclear the focus on IgM responses against DBPII-Sal1 & DEKnull-2 such a long term survey.* (…)

***Specific comment -1*** 1. The authors have to justify (…), *why focus on IgM response against DBPII-Sal1 & DEKnull-2 during such a long 9 year period? (…)Why did authors focus on IgG and IgM responses against DBPII-Sal1 and DEKnull-2? And what is the major new finding on the current study? (…)* In generally, IgM antibodies appear early in the course of an infection and usually reappear, to a lesser extent, after further exposure.(…) And also it is not justified yet to select DBPII-Sal1 & DEKnull-2 antigens for this study due to the authors mentioned these sentence as below (Ref 35) “Thus, while the DEKnull-2 antibody response is not a useful tool for serological evaluation of malaria transmission, it seems to be appropriate to evaluate naturally-acquired immunity

**AUTHORS:** Currently, a significant body of evidence point to an underappreciated role for IgM in protection against infectious disease including malaria; for example:

***(1)*** In experimental malaria, mice lacking specific-IgG antibodies but with strong anti-parasite IgM antibody responses develop long-lasting non-sterile immunity and survive lethal parasite challenge (Borges da Silva et al., 2018; PMID: 30148845);

***(2)*** Somatically hypermutated *Plasmodium-*specific IgM+ MBCs proliferated and gave rise to antibody-secreting cells that dominated the early secondary response to parasite rechallenge (Krishnamurty et al., 2016; PMID:27473412);

***(3)*** IgM antibodies (but in much less extension IgG) to a broad array of *P. falciparum* antigens was associated to genetic resistance to malaria (Arama et al., 2015; PMID: 26361633);

***(4)*** In *P.falciparum* malaria, merozoite-specific IgM is an important functional and long-lived antibody response targeting blood-stage malaria parasites that contributes to malaria immunity (Boyle et al., 2019; PMID: 31579826);

(5) IgM antibodies may be necessary to sustain an optimal long-term protective IgG response (Harte et al., 1983; PMID: 6835362; Boes et al., 1998; PMID: 9590224),

Taken together, *we are in accordance with other that suggest that IgM antibodies seem to be much more than just an early responder to malaria infection (Boonyaratanakornkit & Taylor, 2019; PMID: 31603585), and should be investigated during the development of vaccines.*

In pursuing a *P.vivax* DBPII structural vaccinology approach, we created DEKnull-2 antigens lacking the variant epitopes but retaining the conserved protective functional epitopes, which are essential for binding its cognate reticulocyte receptor. As quoted above, DEKnull-2 should be used to evaluate natural acquired immunity and not fluctuation of *P.vivax* transmission (ref. 35). Considering the new paradigm of the relevance of IgM response in malaria (as summarized above), we sought to investigate here the relationship between the IgM and IgG antibody responses against conserved (DEKnull-2) and variable DBPII epitopes circulating in the area. *In the current study, a comparison between conserved and variable epitopes was critical, as DBPII-based vaccine should target strain-transcending immune responses.* *As IgM antibodies may be necessary to sustain an optimal long-term protective IgG response (PMID: 6835362; PMID: 9590224),* we also investigate whether a stable DBPII-specific IgM response could interfere with the profile of functional antibodies, i.e., antibodies able to block the interaction ligand-receptor (BIAbs).

Here, we demonstrated for the first time that long-term exposure to low and unstable levels of *P. vivax* transmission lead to a sustained DBPII-specific IgM response toward variant-specific epitopes while sustained IgG responses are skewed to conserved epitopes involved in protection (DEKnull-2 over Sal-1). Although a persistent DBPII-specific IgM response was not associated with broadly neutralizing IgG antibody response, future studies should investigate on the role of IgM in the DBPII acquired immune response (perhaps to sustain IgG response). We are confident that these findings will contribute to the current stage of development of DBP-based vaccines that focus broad protective immune response.

*In the revised version of the MS we clarify this topic, as requested to the reviewer (see MS with track changes). More specifically, although most of the references on the importance of studying IgM antibodies were already included in the penultimate paragraph of the introduction, we reinforced this topic in the final paragraphs of the introduction and included additional references.*

Rev#2: And also a significant problem of this manuscript is *figure 1 has been used on previous article*, and it should be removed from main MS or move it on to supplemental figure.

**AUTHORS**: the original Fig 1 was included in the Methods to clarify the study design and the long-term follow up cohort that was carried before (ref35); otherwise, the readers will need to access the ref 35. However, as requested to reviewer, Fig. 1 was included in the revised version of the MS as supplementary (now Fig 1S).

Rev#2: Careful editing will greatly strengthen the manuscript.

**AUTHORS**: The MS was revised by Dr. John H Adams, South Florida University, USA (<https://health.usf.edu/publichealth/overviewcoph/faculty/john-adams>). Dr. Adams is also a co-author of the MS.

**Specific point** 2. It is required the authors that ref 35 has to refer in the introduction on this manuscript, especially it is very important to mention it what they found it before. I assume this MS is a kind of retrospective serological follow up study, due to this, it has to be refereed well all the detail for them previous work. *And also they need to explain the advantage of BIAbs assay due to clarify for the difference from Ref 35*.

**AUTHORS**: As we clarified above (please see major comments) the MS from Pires et al (ref 35) was design to evaluate seroconversion rates as a tool for assess trends in *P.vivax* malaria transmission; in that study to evaluate the breadth of antibody response was critical to included antigens covering a wide range of immunogenicity (MSP1-19, AMA-1 and DBPIIs). On the other hand, the current MS was design to get insides in the acquired immune response to a novel engineered vaccine candidate - DEKnull-2. As quoted before, we have included in the last paragraph of the introduction that we took advantage of the long-term follow-up study previously carried out in the Amazon rainforest, where different profiles of DBPII-specific IgG responders were identified (Pires et al., 2018). *In the current MS, it was critical to evaluate BIABs response because a strong naturally acquired BIAb response is associated with a reduced risk of clinical P. vivax malaria* (King et al., 2008; PMID: 18523022; Nicolete et al., 2016; PMID: 27578850). All these references were included in the MS (please see highlighted text in the 2^nd^ paragraph of the introduction, track changes MS).

**Specific point** 3. Fig 1 had been used on them previous article (Ref 35, fig1), and then it has to remove from main MS or move it on to supplemental figure. (Such a minor change will not be called as a “modified”)

**AUTHORS**: Okay, it was done. In the revised version of the MS, Fig 1 was included as supplementary Fig S1

**Specific point** 4. Fig 2 and 3 have to remove from main MS or move it on to supplemental figure. These figures are just raw data sets for fig 4.

**AUTHORS**: Okay, it was done. Figures were adjusted as suggested (Fig S2 and S3)

**Specific point 5.** The information of right panel of fig 5 is not founded on the current manuscript. (…)

**AUTHORS**: Perhaps the results in Fig. 5 have led to a misunderstanding. More specifically, Fig 5 shows every single individual who had blood-stage infection at any time of the follow-up study (left panel), and in the right panel (the heatmap) their correspondent antibody responses to DBPII-related antigens, including both IgM (1^st^ part of the right panel, i.e., the first part of the heatmap) and IgG antibodies (2^nd^ part of heatmap). At the top of heat map, IgM and IgG antibodies were identified in bold. The results showed no correlation between acute infection and either antigen-specific IgM or IgG antibodies. As example, in the text, we have identified some specific individuals (by code) and comments about their results in the MS. At this time, we should clarify that all data from the original Fig. 5 (now Fig. 2 as requested by the reviewer) were included in the text and/or in the legend of the figure (please see highlighted text in the track changes copy).

Rev#2: (…) What is the IR? (…)

In the figure, the results of antibody response were expressed as the ELISA reactivity index (IR) that was calculated as described in the methods (please see highlighted text in the item IgM and IgG detection assays). Briefly, the ELISA reactivity (RI) was calculated as the ratio of the mean optical density (OD at 492 nm) of each sample to the mean OD plus three standard deviations of samples from 20-30 unexposed volunteers. Values of RI > 1.0 were considered positive (on a green scale). In the revised version of the MS, we included RI definition in the legend of Fig 2 (ex-Fig 5).

Rev#2:The reviewer would not be founded any advantage points about this figure except left part.

AUTHORS: We are confident about the relevance of the data presented in the figure 5, particularly, due to the absence of correlation between antigen-specific IgM antibodies and acute malaria infection. These findings are intriguing as the antibodies made initially against infection are usually IgM. Perhaps a plausible explanation is the relative underrepresentation of *P. vivax* asexual stages in patient blood as a considerable body of evidence indicates a tissue reservoir, such as bone marrow, where most *P. vivax* parasite burden resides (please see 2^nd^ paragraph of discussion). Consequently, we decided to keep figure 5 (now retitled as Fig. 2).

Rev #2: Moreover it is useful to combine the Pv-infected information (fig 5 left panel) with figure 6. Taken together, it would be getting better to merge fig 5 and 6.

AUTHORS: While the figure 5 shows the absence of correction between antigen-specific antibody responses and acute malaria infection, Fig 6 shows the IgM/IgG antibody responses according to the profile of binding inhibitory activity (BIAbs) as detected by functional assays (COS-7 transfected cells). Consequently, it is not possible to merge results that are unrelated.

We would like to thank deeply the reviewer for his time and effort, and we hope that the MS is now suitable for publication.
